# Age-Related Changes in Task Related Functional Network Connectivity

**DOI:** 10.1371/journal.pone.0044421

**Published:** 2012-09-18

**Authors:** Jason Steffener, Christian G. Habeck, Yaakov Stern

**Affiliations:** Cognitive Neuroscience Division, Department of Neurology and Taub Institute for Research on Alzheimer's Disease and the Aging Brain, Columbia University College of Physicians and Surgeons, New York, New York, United States of America; Beijing Normal University, China

## Abstract

Aging has a multi-faceted impact on brain structure, brain function and cognitive task performance, but the interaction of these different age-related changes is largely unexplored. We hypothesize that age-related structural changes alter the functional connectivity within the brain, resulting in altered task performance during cognitive challenges. In this neuroimaging study, we used independent components analysis to identify spatial patterns of coordinated functional activity involved in the performance of a verbal delayed item recognition task from 75 healthy young and 37 healthy old adults. Strength of functional connectivity between spatial components was assessed for age group differences and related to speeded task performance. We then assessed whether age-related differences in global brain volume were associated with age-related differences in functional network connectivity. Both age groups used a series of spatial components during the verbal working memory task and the strength and distribution of functional network connectivity between these components differed across the age groups. Poorer task performance, i.e. slower speed with increasing memory load, in the old adults was associated with decreases in functional network connectivity between components comprised of the supplementary motor area and the middle cingulate and between the precuneus and the middle/superior frontal cortex. Advancing age also led to decreased brain volume; however, there was no evidence to support the hypothesis that age-related alterations in functional network connectivity were the result of global brain volume changes. These results suggest that age-related differences in the coordination of neural activity between brain regions partially underlie differences in cognitive performance.

## Introduction

Advancing age is associated with decline in multiple cognitive domains, including verbal working memory (WM). Verbal working memory describes the maintenance and manipulation of verbal information over the course of several seconds [1]. Encoding, maintaining and retrieving information using verbal working memory involves the coordination of multiple neural processes. The brain regions sub-serving these processes have been elucidated with experimental manipulations that vary task demands by altering the information load [2], [3], [4], retention interval [5], [6] and response time duration [7], [8]. Results from fMRI studies largely demonstrate increased signal within the pre-frontal, premotor and parietal cortices with these alterations in task demands [9].

Advancing age is associated with decreased performance on verbal working memory in the face of increasing task demands [10]. The underlying cause for these cognitive changes is assumed to partly be age-related neural changes [11] in global and regional brain volume, white matter hyperintensity burden and cerebral blood flow. More recently, aging and disease have also been related to changes in the functional connectivity of brain regions in the absence of external stimulation [12], [13], [14], [15].

The present study examines how the strength of functional connectivity between broad networks of brain regions during performance of a verbal working memory task relates to task performance. Measures of functional network connectivity characterize within-participant neural interactions between functionally interconnected networks of brain regions [16]. This approach investigates how networks of brain regions influence other networks of brain regions in the face of age-related changes. The mechanisms underlying changes in functional network connectivity could indicate abnormal effects that consistently affect multiple brain structures, such as abnormal inter-cellular signaling [17]. Furthermore, the identified networks respect functional boundaries within participants, which is not necessarily true using anatomical defined regions of interest [18]. Additionally, measures of connectivity are robust against many of the age-related effects that may weaken the relationship between statistical parametric models (SPM) of the task and the BOLD response in older adults [19]. Age-related decreases in the coordination of brain regions may mediate, or underlie, the age-related performance changes seen on cognitive tasks. If advancing age affects the strength of functional network connectivity between brain regions during task performance, the question arises as to whether the underlying brain structure is the cause of such dis-coordination. We hypothesize that advancing age is associated with brain volume changes, strength of functional network connectivity between networks and declines in task performance. Furthermore, we hypothesize that these age-related changes form a causal chain such that task performance decline is due to changes in functional network connectivity, which results from age-related brain volume changes. The current work aims to determine if there is evidence to support these ideas by addressing three questions:

1. Does aging change the functional network connectivity between brain regions during performance of a verbal working memory task?
2. Are age-related changes in task performance associated with changes in functional network connectivity?
3. Are changes in functional network connectivity associated with changes in global brain volume?

These questions were addressed using an independent components analysis (ICA) of fMRI data collected from young and old healthy adults while performing a verbal delayed item recognition (DIR) task and a subsequent series of mediation analyses. The ICA identified maximally spatially independent brain maps such that the brain regions included in each map share similar time-courses within each participant and respect individual functional boundaries [17]. Once the ICs were identified, the degree to which an individual utilized each map over time was compared to the modeled time course of the task itself. This allows classification of ICs as being task related or not task related. The time courses of each IC were then correlated with one another within each participant, providing measures of the strength of the functional connectivity between the ICs [16]. We then tested for age group differences in the strength of the functional connectivity between ICs. Based on studies of the default mode network we expected decreased strength of correlation between functional networks with advancing age [20] that would affect task performance [21]. Specifically, we expected anterior to posterior functional connections to be disrupted by aging [12] as well as sub-cortical connections, whose strength have been shown to affect verbal memory [13]. Therefore, we additionally examined whether the effect of advanced age on task performance was mediated by the functional connectivity across ICs. Finally, we examined whether age-related changes in whole brain volume could explain any age-related changes in functional connectivity. The results from these analyses are discussed within the context of a recently presented conceptual model of advancing age [22], [23].

## Methods

### Study Participants

The current study used data from 75 healthy, young participants (50 men and 25 women mean (±s.d.) age = 24.45 (3.50); mean (± s.d.) years of education = 15.72±1.50; all right handed), and 37 healthy, old participants (15 men and 22 women; mean (± s.d.) age = 71.27±6.29; mean (± s.d.) years of education = 16.32±2.58; all right handed). Written informed consent was obtained from all participants under a protocol approved by the Internal Review Board of Columbia University. This sample is a complete collection of young and old adults who have participated in studies at our facility on the same DIR task, on the same scanner and previous analyses on subgroups have been published [3], [4], [24], [25], [26]. All participants were screened with structured medical, neurological, psychiatric, and neuropsychological evaluations to ensure that they had no neurological or psychiatric disease or cognitive impairment. The screening procedure included a detailed interview that excluded individuals with a self-reported history of major or unstable medical illness, significant neurological history (e.g. epilepsy, brain tumor, stroke), history of head trauma with loss of consciousness for greater than 5 min or history of Axis I psychiatric disorder [27]. Individuals taking psychotropic medications were also excluded. Global cognitive functioning was assessed with a modified version of the Folstein Mini Mental State Examination (mMMS: [28]), which has a maximum score of 57. All participants were classified as non-demented and without clinically significant cognitive impairment and although they differed in the mMMS, (young mean (± s.d.) mMMS total = 55.19±1.82; old mean (± s.d.) mMMS total = 53.58±3.06, *t* (51.66) = 2.83, *p* = .007)) this difference was not clinically significant. IQ was estimated with the American version of the New Adult Reading Test (NART: [29]). The old NART scores were not significantly lower than the young scores (young mean (± s.d.) NARTIQ = 119.151±6.22, old mean (± s.d.) NARTIQ = 117.40±7.51; *t* (106) = 1.56, *p* = .12) and were above average for both groups.

### Behavioral Task

All participants performed a verbal delayed item recognition task in which memory load was manipulated by the number of letters (1, 3, or 6) the subject needed to store in working memory [2], [10]. Task parameters and training procedures were identical to those reported in our previous studies [3], [30]. In brief, each trial of the delayed item recognition task consisted of a stimulus presentation, retention delay, and probe presentation. Stimulus set size was pseudo-randomly varied across trials and each of the three experimental runs contained 10 trials at each of the three memory load levels, with five match trials and five non-match trials. In all, there were 30 trials per set size per participant. Time between trials varied, producing a jittered inter-trial interval length to reduce anticipatory effects and prevent MRI scanning to be time-locked to the hemodynamics [31]. Participants indicated whether the probe item was included in the initial set by a differential button press (left hand = no, right hand = yes) and were instructed to respond as quickly as possible. Median response times and accuracy, independent of response bias (d_L_), for each memory load level were used as behavioral measures.

### MRI data acquisition

During performance of the three task blocks 207 T2*-weighted BOLD images, were acquired with an Intera 1.5 Tesla Philips MR scanner equipped with a standard quadrature head coil, using a gradient echo echo-planar (GE-EPI) sequence (TE/TR = 50 ms/3000 ms; flip angle = 90°; 64×64 matrix, in-plane voxel size = 3.124 mm×3.124 mm; slice thickness = 8 mm (no gap); 17 trans-axial slices per volume). Four additional GE-EPI excitations were performed before the task began to allow transverse magnetization immediately after radio-frequency excitation to approach its steady-state value; the image data for these excitations were discarded. A T1-weighted spoiled gradient image was also acquired from each subject for spatial normalization purposes (TE/TR = 3/25 ms, 256×256 matrix; FOV = 230×186×230 mm; 124 slices per volume).

Task stimuli were back-projected onto a screen located at the foot of the MRI bed using an LCD projector. Participants viewed the screen via a mirror system located in the head coil and had vision corrected to normal as needed using MR compatible glasses (manufactured by SafeVision, LLC. Webster Groves, MO). Responses were made on a LUMItouch response system (Photon Control Company) using the index fingers of either hand. Task administration and collection of RT and accuracy data were controlled using PsyScope 1.2.5 [32] running on a Macintosh G3 iBook. Task onset was electronically synchronized with the MRI acquisition computer. A Carnegie Mellon Button Box (New Micros, Inc. Dallas, TX) provided digital input-output for the response system and synchronization with the MRI acquisition computer, as well as millisecond accurate timing of responses.

### Functional MRI data analysis

All image pre-processing and statistical analyses used SPM5 (Wellcome Department of Cognitive Neurology). For each subject's EPI dataset: images were temporally shifted to correct for slice acquisition order using the first slice acquired in the TR as the reference. All EPI images were corrected for motion by realigning to the first volume of the first session. The T1-weighted (structural) image was co-registered to the first EPI volume using mutual information. This co-registered high-resolution image was used to determine the transformation into a standard space defined by the Montreal Neurologic Institute (MNI) template brain supplied with SPM5. This transformation was applied to the EPI data and re-sliced using sync-interpolation to 2×2×2 mm. Finally, all images were spatially smoothed with an 8 mm FWHM kernel.

### Time series models

The time series models crossed the load (1, 3 or 6 letters) and task phase (stimulus, retention and recognition) factors to create nine regressors of interest. The stimulus phase for each load level was modeled with a 3 second rectangular epoch, the retention phase for each load level was modeled with a 7-second epoch and the recognition phase for each load level was modeled as rectangular epochs lasting until the trial specific response (i.e. the RT) was made [33]. Trials without motor responses from the participant during the 3-second recognition period (time outs) or where an incorrect response was made were modeled separately, and were not included in any higher-level analyses. All regressors of the time series models were convolved with a standard double-Gamma model of the hemodynamic response function [34]. Contrasts of interest tested for load-dependent and load-independent effects within each task phase resulting in six effects of interest.

### Independent components analysis

Group spatial independent components analysis was applied using the infomax algorithm [35] as implemented in the GIFT software (http://icatb.sourceforge.net/, version 2.0d) and data were decomposed into 36 components as determined by the minimum description length criteria [36]. This approach first used two data reduction steps. The first performed a principal components analysis within each of the three runs within each participant and retained 30 PCs per run. The second data reduction step performed principal components analysis across the 90 retained PCs from all participants and retained 36 PCs. The ICA was performed on these 36 PCs using the ICASSO approach with 20 repetitions [37]. Therefore, the ICA decomposition was repeated 20 times to identify reliable and stable components. This helps ensure that the iterative ICA procedure identifies global minima and not local minima. Visual inspection of the components identified artifacts related to eye movements, head motion or pulsations at the base of the brain and removed them from further analyses. Time-courses for each component for each participant were computed via back-reconstruction [38] and the time components were tested for relationships to the task using the time series model described above using general linear modeling analyses. Contrasts testing for load-dependent and independent signal change within each phase of the task were calculated. The resultant contrast weighted beta-values were tested with one-sample within group *t*-tests controlling for gender. This procedure is analogous to first and second level statistical modeling with one time-course for each component. A component was deemed task-related if it was significantly different from zero in at least one of the two age groups using α = 0.05, Bonferroni corrected for multiple comparisons, controlling for gender.

### Functional network connectivity

Functional network connectivity was calculated as the Pearson correlation coefficient between the time courses for each component within each participant. The resultant coefficients were Fisher z-transformed to match normally distributed values. Tests of the measures of functional network connectivity determined if age group affected their strength. Between age group two-sample *t*-tests determined whether the strength of functional network connectivity between spatial components differed between the age groups using α = 0.05, Bonferroni corrected for multiple comparisons, controlling for gender.

### Functional network connectivity and task performance

Linear regression tested the relationship between the strength of functional network connectivity between components and task performance while controlling for gender. An alpha value of 0.05 Bonferroni corrected for multiple comparisons was used.

### Functional network connectivity mediation

**Figure 1:**
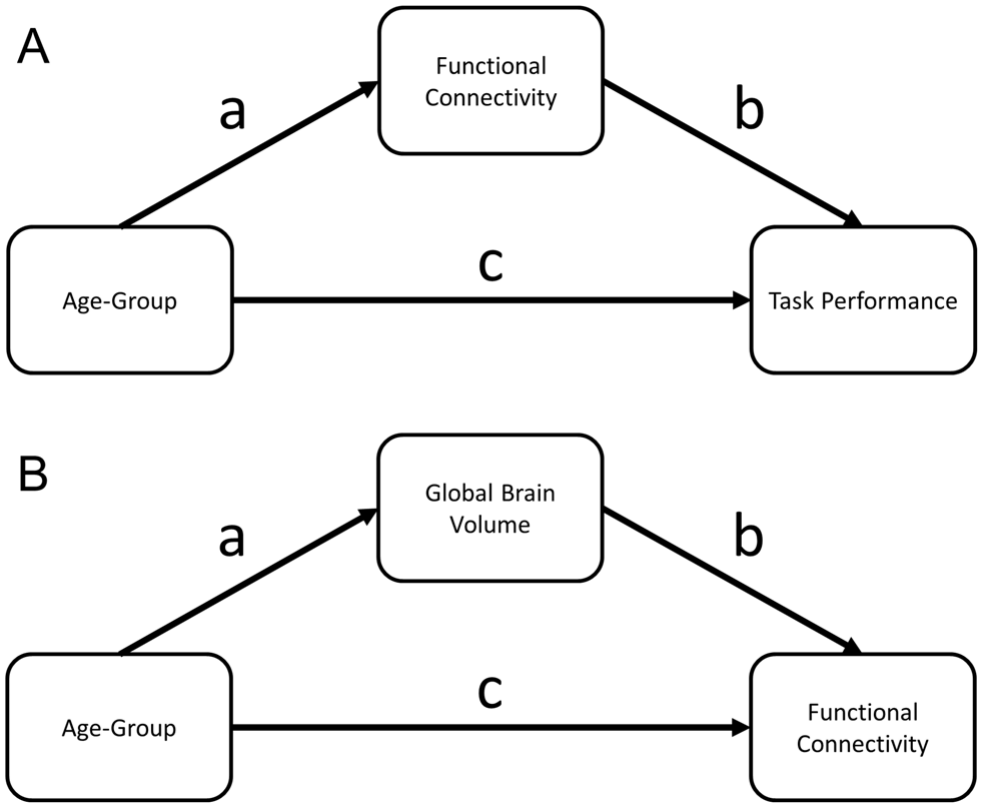
The mediation models. A) Model testing whether functional network connectivity is in the causal pathway between age-group and task performance. B) Model testing whether global brain volume is in the causal pathway between age-group and functional network connectivity.

Mediation tests determined whether the effect of advanced age on task performance was due to age related changes in functional network connectivity, see Figure 1A. Findings of a significant indirect effect between age group and task performance via functional network connectivity are consistent with the causal chain that assumes advancing age has an effect on functional network connectivity, which affects task performance. The mediation analysis tested this indirect effect while controlling for gender using 10,000 stratified bootstrap resamples to determine the bias-corrected percentile confidence intervals using the Mediate SPSS tool [39], [40], [41], [42]. Age group is a categorical variable and the stratified bootstrapping procedure preserves sample sizes in each age group avoiding bias in the resamples due to the different sample sizes in the age groups.

### Brain volume mediation

Mediation tests determined whether the effect of advanced age on functional network connectivity resulted from changes in a global brain structure, see Figure 1B. Normalized whole brain volume (nWBV) was used as a measure of brain structure and is calculated from the image segments in native space as the summed volume of gray- and white-matter divided by the summed volume of gray-matter, white-matter and cerebrospinal fluid [43]. This measure represents the percentage of the total intracranial volume occupied by gray and white matter and provides a simple summary measure of age-related loss of gray and white cerebral tissue volume. This mediation analysis tested the indirect effect that age has on functional network connectivity through nWBV while controlling for gender using 10,000 stratified bootstrap resamples to determine the bias-corrected percentile confidence intervals. Age group was a categorical variable using stratified bootstrap resampling.

## Results

### Task performance

Increased task demands increased response times for both age groups (Table 1). Using a repeated measures design, and Huynh-Feldt correction for non-sphericity, response time was affected by letter set size while controlling for gender in both young (*F*(1.75,127.52) = 63.50, *p*<0.001, η^2^ = 0.46) and old (1.29, 45.15) = 61.70, *p*<0.001, η^2^ = 0.64) groups. There was no interaction between gender and load for the young (*F*(1.75,127.52) = 0.26, *p*>0.05, η^2^ = 0.003) nor the old (*F*(1.29, 45.17) = 0.062, *p*>0.05, η^2^ = 0.002) groups. The interaction of age group and set size was also significant (*F*(1.55, 167.56) = 4.94, *p*<0.05, η^2^ = 0.044), the interaction between set size and gender was not significant (*F*(1.55,167.56) = 0.20, *p*>0.05, η^2^ = 0.002), nor was the interaction between set size, age group and gender (*F*(1.55, 167.56) = 0.051, *p*>0.05, η^2^ = 0.000). Based on this letter set size related finding, the letter set size dependent effect (slope) on response times (sRT) was calculated and was significantly larger in the older adults than the young (*F*(1,108) = 14.38, two-tailed *p*<0.001), there was no main effect of gender (*F*(1,108) = 0.013, *p*>0.05), nor an interaction of gender and age group (*F*(1,108) = 0.059, *p*>0.05).

**Table 1:** Mean (standard deviation) response times and accuracies (d_L_).

	Response Time		Accuracy	
	Young	Elder	Young	Elder
1 letter	0.88(0.19)	0.90(0.19)	2.54(0.49)	2.34(0.50)
3 letters	1.01(0.22)	1.09(0.21)	2.51(0.52)	2.32(0.45)
6 letters	1.18(0.26)	1.35(0.32)	2.48(0.60)	2.40(0.57)

Accuracy independent of response bias (d_L_) was not affected by set size in the young (*F*(2.00, 146.00) = 1.14, *p*>0.05, η^2^ = 0.015); however, there was an interaction between set size and gender (*F*(2,146) = 5.77, *p*<0.01, η^2^ = 0.073). The elder participants had no effect of set size (*F*(2.00, 70.00) = 0.18, *p*>0.05, η^2^ = 0.00), nor any significant interactions with gender. The interaction of age group and set size was not significant (*F*(2.00, 216.00) = 0.61, *p*>0.05, η^2^ = 0.006); however, the interaction between set size and gender was (*F*(2, 216) = 4.44, *p*<0.05, η^2^ = 0.04).

### fMRI results

**Figure 2:**
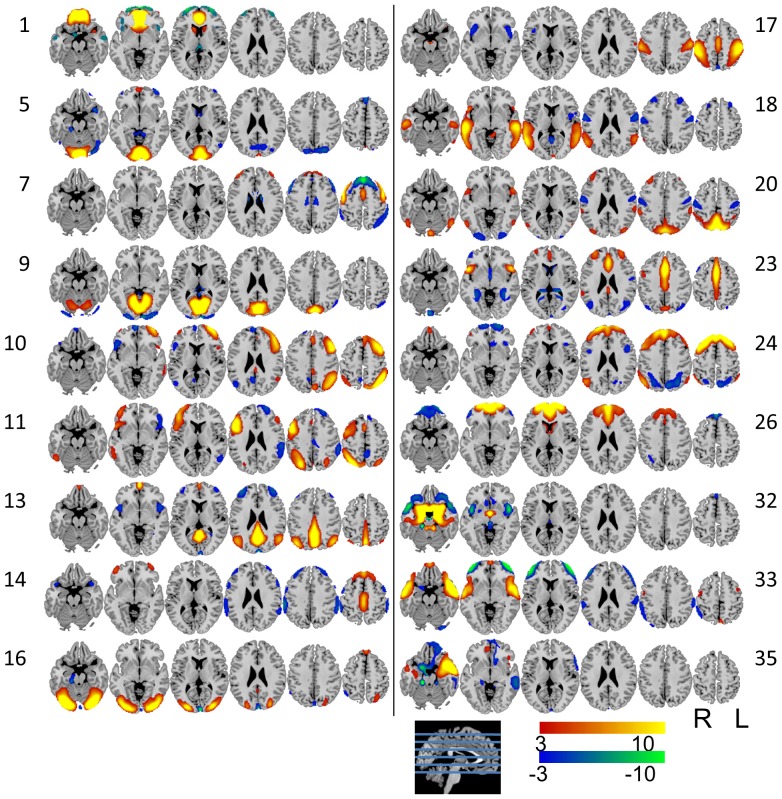
The eighteen spatially independent task-related components. The components are numbered using the arbitrary ordering resulting from the ICA which extracted 36 total components.

Of the original 36 independent components, 11 were identified as artifacts (Figure S1 in Supporting Information S1) and 7 were not related to any aspect of the task for either age group (Figure S2 in Supporting Information S1) leaving 18 components (see Tables 2 and 3). Figure 2 shows the 18 components and Tables S1 through S18 in Supporting Information S1 summarize the component maps with Brodmann areas (BA) and regions of activation. The ICs are very briefly described here based on their largest sources and are numbered according to their arbitrary order from the ICA: 1) bilateral middle frontal orbit; 5) bilateral calcarine; 7) bilateral supplementary motor area, precentral; 9) bilateral calcarine; 10) left inferior parietal, middle frontal; 11) right inferior parietal, middle frontal; 13) bilateral precuneus, angular; 14) bilateral medial frontal, cingulate; 16) bilateral cerebellum, lingual; 17) bilateral post-central; 18) bilateral middle temporal; 20) bilateral precuneus; 23) bilateral middle cingulate; 24) bilateral medial superior frontal; 26) bilateral medial superior frontal; 32) bilateral hippocampus; 33) bilateral middle temporal and 35) left middle temporal pole.

**Table 2:** Within and between age group comparisons of the individual time-course of each component and the load dependent contrasts for each task phase.

	Load-dependent								
	Stimulus			Retention			Probe		
	Young	Elder	Y>E	Young	Elder	Y>E	Young	Elder	Y>E
1	−3.49	−0.80	−0.96	−5.35**	−3.49	−2.18	−3.94	0.22	1.05
5	7.74**	2.46	−1.48	2.69	1.44	1.49	−0.26	−1.53	0.39
7	3.97	3.67	0.02	4.35**	4.60**	3.45	1.74	0.4	−1.06
9	4.70**	4.20	1.13	−0.90	1.95	2.87	−0.74	−1.4	−1.42
10	4.33**	3.06	1.15	4.35**	4.74**	3.17	0.09	2.21	0.91
11	6.33**	3.08	0.01	7.07**	6.98**	1.89	3.12	2.25	−1.09
13	−5.05**	−2.63	0.66	−6.89**	−3.42	0.58	−3.41	−1.60	0.86
14	−2.12	0.01	1.29	−1.52	0.52	2.25	−0.79	−0.54	0.17
16	8.43**	5.50**	2.08	1.09	3.15	3.97	−2.40	−2.23	−0.09
17	1.21	2.55	1.52	3.04	2.66	3.48	−1.28	0.76	0.03
18	−5.45**	−2.41	−0.77	−5.38**	−2.36	−0.20	−0.55	0.68	0.98
20	1.69	2.21	2.48	1.50	5.78**	5.63**	−0.75	0.44	0.16
23	4.48**	2.83	0.31	3.43	3.96	0.91	0.69	−0.96	−1.60
24	−5.95**	−4.71**	−2.17	−5.51**	−3.66	−1.99	−1.14	1.20	1.34
26	−4.42**	−2.62	−1.9	−6.60**	−1.57	0.36	−1.96	−0.46	−0.07
32	−0.78	−3.21	−1.68	1.39	−0.48	−0.2	0.85	−0.55	−0.28
33	1.44	1.38	1.83	−0.46	1.30	0.34	−0.12	1.19	1.58
35	−3.39	−2.94	−1.32	−2.04	−2.86	−1.22	−1.52	−2.72	−0.96

Notes:

** *p*<0.01 corrected for 300 multiple comparisons. All measures represent *t*-values when testing for differences from 0 for within group measures and differences between groups for those comparisons.

**Table 3:** Within and between age group comparisons of the individual time-course of each component and the load independent contrasts for each task phase.

	Load-independent								
	Stimulus			Retention			Probe		
	Young	Elder	Y>E	Young	Elder	Y>E	Young	Elder	Y>E
1	−2.94	−4.36	−1.84	−3.11	−1.66	−0.91	−4.31**	−4.35	1.16
5	10.70**	3.61	−1.97	−1.79	−2.18	0.22	−0.02	0.00	−0.16
7	7.55**	4.33	0.60	2.22	3.80	0.79	5.12**	2.34	−1.73
9	5.77**	2.44	−0.06	−5.57**	−3.30	0.83	2.51	−1.00	−2.31
10	4.97**	3.95	2.55	−0.51	2.51	2.24	5.83**	2.27	−1.27
11	9.35**	6.76**	2.56	6.58**	5.83**	1.48	11.25**	6.88**	−0.70
13	−5.55**	−4.3	−0.84	−4.24**	−3.60	−0.38	−2.17	−3.44	−0.82
14	−2.85	−0.72	1.09	−0.88	0.25	0.76	−4.51**	1.18	3.47
16	10.54**	6.39**	0.40	−3.88	−2.71	1.73	5.12**	3.56	−1.76
17	5.07**	2.38	0.96	0.93	2.45	1.62	6.49**	4.02	−0.12
18	−6.36**	−3.77	−0.14	−3.80	−1.98	−0.62	−0.86	−3.89	−3.50
20	7.53**	2.61	2.10	−0.47	2.20	2.06	2.11	1.51	0.87
23	4.97**	2.90	−0.51	3.46	4.22	1.23	8.73**	5.92**	−3.03
24	−9.93**	−8.00**	−2.89	−4.54**	−2.07	−0.33	−6.39**	−6.81**	−0.12
26	−7.75**	−4.51**	−0.89	−3.63	−1.15	−0.97	−4.95**	−4.41	−1.51
32	1.84	−0.44	−1.72	2.66	0.75	−0.20	−6.36**	0.14	3.88
33	1.53	−1.70	−1.56	−0.37	−1.74	0.48	−5.11**	−0.89	2.75
35	−7.28**	−1.65	0.62	−1.08	−3.38	−2.00	−2.69	0.74	2.31

Notes:

** *p*<0.01 corrected for 300 multiple comparisons. All measures represent *t*-values when testing for differences from 0 for within group measures and differences between groups for those comparisons.

### Does age affect the functional network connectivity between brain regions during a cognitive challenge?

**Figure 3:**
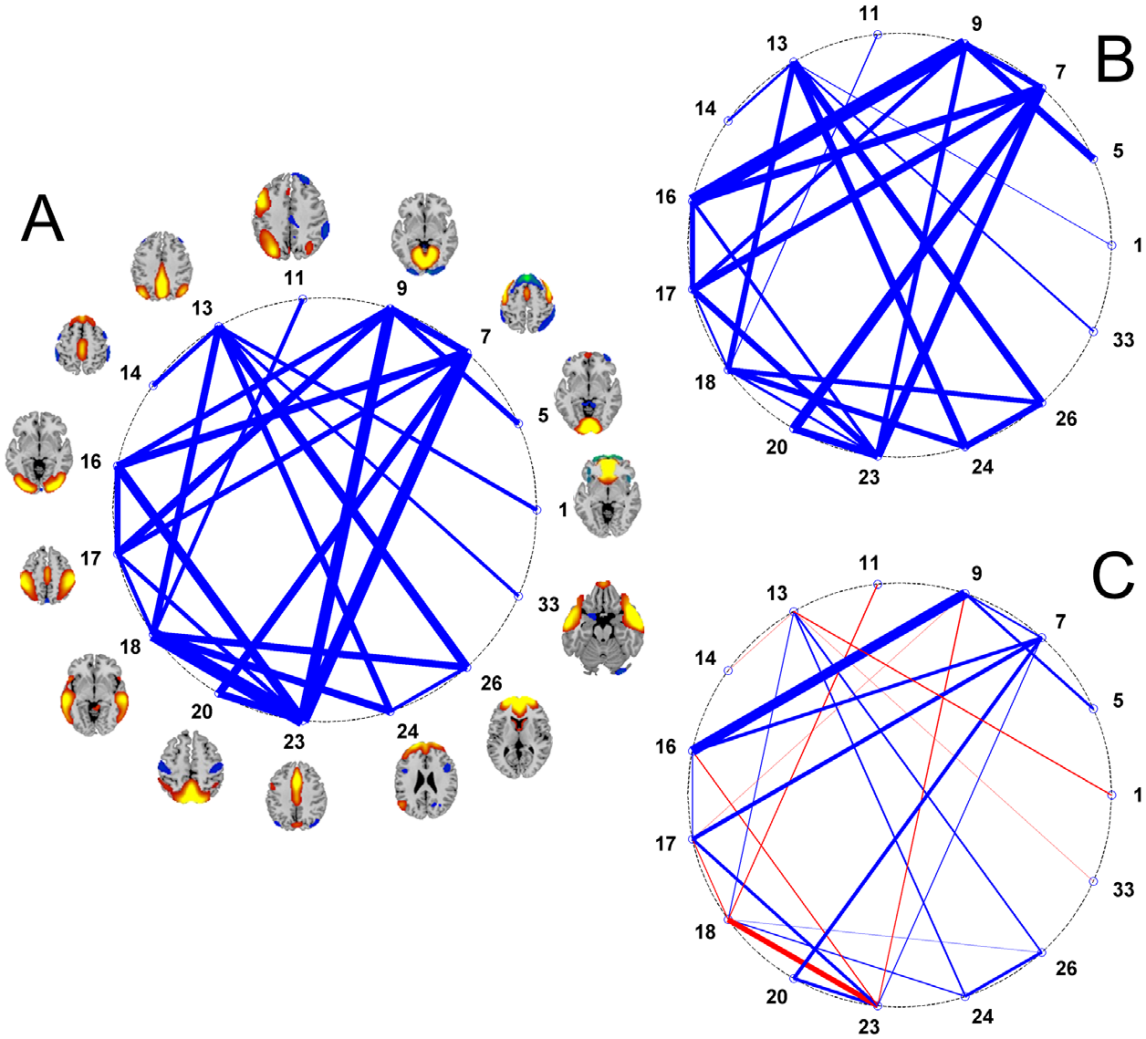
The functional network connectivity between 15 spatially independent task-related components. A) The lines represent the between age group *t*-values of functional network connectivity. The mean functional network connectivity for the young B) and old C) age groups is presented for descriptive purposes showing the functional network connections that significantly differ between age groups. The placement of the nodes around the circles is related to the order in which the components were extracted with ICA. Therefore, the placement is arbitrary and the reader should not make any inference on the length of the edges in the graphs, only on the fact that the functional network connections are for the most part stronger in the young than the elder age groups.

**Figure 4:**
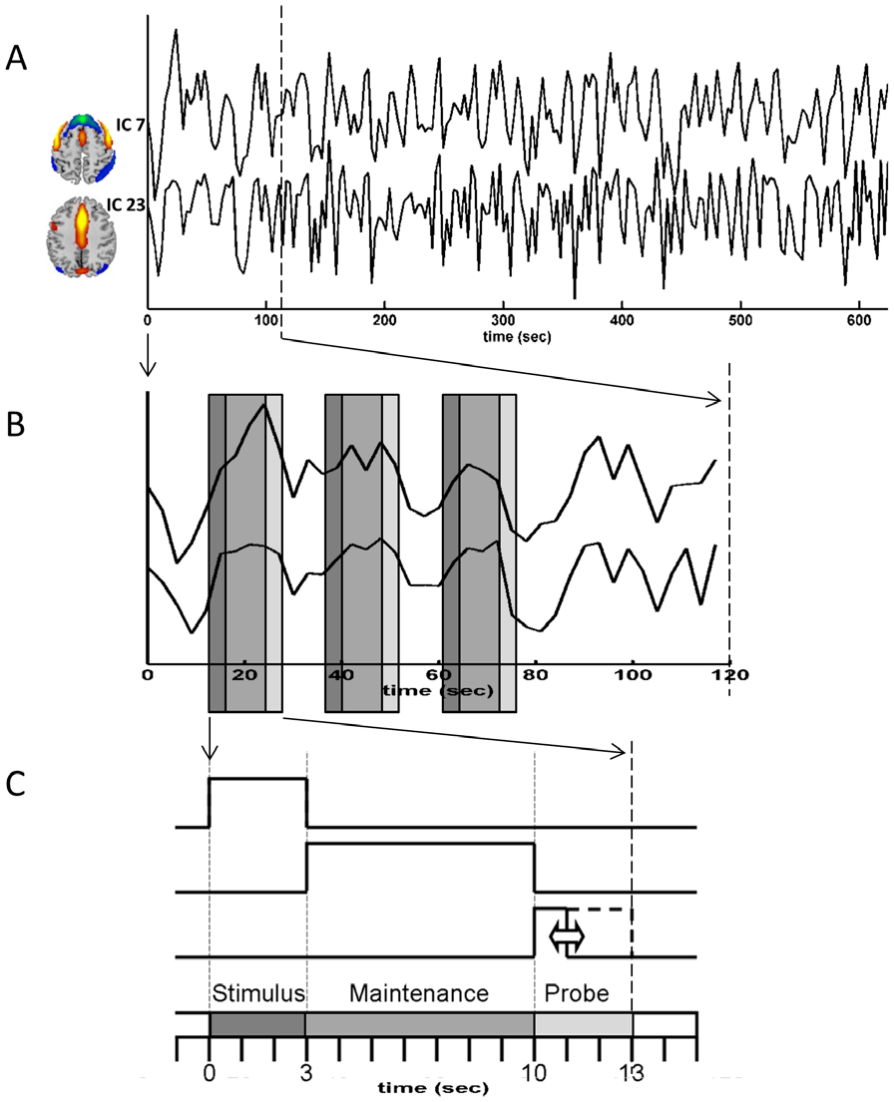
Example time courses and their relationship with the task. A) Example time courses from independent components 7 and 23 from a single participant. The correlation between these two time courses is *r* = 0.65. B) A zoom in on the first 120 seconds of the two time courses. The gray underlying bars represent the three task phases. C) A zoom in on one trial shows the three task phases: stimulus, maintenance and probe.

Significant between group differences in the strength of the functional network connectivity between ICs were always the result of the young age group being larger than the older adults (see Figure 3 and Table 4). Three components (10, 32, 35) had no significant between group differences in functional network connectivity with any of the other ICs and were removed from further analyses. Figure 4 exemplifies the relationship between two components and the different task phases of the trials.

**Table 4:** T-test results for group differences in strength of functional network connectivity between components and the mean and standard errors for each group.

	t (Y>E)	Young Mean (s.e.)	Elder Mean (s.e.)
Comp 1–13	4.092**	0.065(0.021)	−0.086(0.030)
Comp 5–9	4.796**	0.424(0.033)	0.170(0.035)
Comp 7–9	5.554**	0.344(0.024)	0.114(0.034)
Comp 7–16	6.109**	0.462(0.028)	0.194(0.028)
Comp 7–17	4.886**	0.461(0.026)	0.234(0.040)
Comp 7–20	6.028**	0.573(0.033)	0.220(0.050)
Comp 7–23	7.906**	0.514(0.034)	0.059(0.044)
Comp 9–16	5.108**	0.773(0.026)	0.560(0.029)
Comp 9–17	5.942**	0.282(0.030)	−0.014(0.038)
Comp 9–23	7.561**	0.313(0.027)	−0.077(0.049)
Comp 11–18	4.003**	0.095(0.026)	−0.082(0.034)
Comp 13–14	4.440**	0.172(0.022)	−0.004(0.035)
Comp 13–18	5.566**	0.327(0.029)	0.056(0.037)
Comp 13–24	5.289**	0.448(0.037)	0.113(0.048)
Comp 13–26	6.389**	0.458(0.034)	0.100(0.040)
Comp 13–33	3.747**	0.121(0.022)	−0.028(0.036)
Comp 16–17	5.050**	0.302(0.029)	0.069(0.031)
Comp 16–23	6.453**	0.220(0.027)	−0.096(0.042)
Comp 17–18	4.018**	0.132(0.030)	−0.072(0.037)
Comp 17–23	4.164**	0.375(0.021)	0.191(0.047)
Comp 18–23	8.860**	0.143(0.029)	−0.311(0.042)
Comp 18–24	5.778**	0.359(0.029)	0.078(0.037)
Comp 18–26	5.463**	0.290(0.024)	0.036(0.046)
Comp 20–23	5.556**	0.420(0.025)	0.175(0.037)
Comp 24–26	3.881**	0.400(0.035)	0.174(0.041)

All measures represent *t*-values when testing for differences from 0 for within group measures and differences between groups for those comparisons.

Notes:

** *p*<0.01 corrected for 153 multiple comparisons.

### Are age-related changes in task performance associated with changes in functional network connectivity?

The strength of functional network connectivity between ICs 7 & 23 and 13 & 24, demonstrated significant relationships with the sRT measure of task performance. Regression analyses demonstrated that all paths between age group, functional network connectivity and task performance were significant. Both of the functional network connections were also significant mediators in the relationship between age group and task performance as demonstrated by significant indirect effects, (Table 5). Although the three measures in each model are significantly related, the significant indirect test supports the hypothesis that the effect of advancing age on declining task performance is partially the result of changes in functional network connectivity.

**Table 5:** Mediation analyses for the relationships of age group on task performance via the strength of functional network connectivity between independent components.

Mediator	c	a	b	c′	Indirect effects (95% CI)
r(7,23)	0.11*	−0.43*	−0.15*	0.059	0.047 (0.011–0.088)*
r(13,24)	0.11*	−0.30*	−0.14*	0.076*	0.030 (0.0080–0.061)*

Notes: c = total effect of age group on speeded task performance; a = effect of age group on mediator; b = relationship between mediator and speeded task performance; c′ = direct effect of age group on speeded task performance; CI = 95% bootstrap confidence interval for the indirect effect (10,000 stratified resamples);

* *p*<0.05; all tests corrected for gender.

### Are age-related changes in functional network connectivity associated with changes in measures of global brain volume?

Regression analyses indicated that all paths between age group, functional network connectivity between ICs 7 & 23 and 13 & 24 and nWBV were significant. Mediation results show that the effect of age group on the functional connections was not mediated by normalized whole brain volume, (Table 6).

**Table 6:** Mediation analyses for the relationships of age group on the strength of functional network connectivity between independent components via global brain volume.

Outcome	c	a	b	c′	Indirect effects (95% CI)
r(7,23)	−0.43*	−0.14*	−0.44*	−0.37*	−0.066 (−0.24–0.11)
r(13,24)	−0.30*	−0.14*	−0.30*	−0.25*	−0.055 (−0.24–0.15)

Notes: c = total effect of age group on outcome measure of functional network connectivity; a = effect of age group on normalized whole brain volume mediator; b = relationship between normalized whole brain volume mediator and outcome measure of functional network connectivity; c′ = direct effect of age group on outcome measure of functional network connectivity; CI = 95% bootstrap confidence interval for the indirect effect (10,000 stratified resamples);

* *p*<0.05; all tests corrected for gender.

## Discussion

This study demonstrated that advancing age is associated with changes in the functional connectivity between networks of brain regions engaged during performance of a verbal working memory task. These age-related differences in functional network connectivity partially explained significant age group differences in the speeded task performance. In addition, the age-related changes in functional network connectivity were not mediated by differences in global brain volume.

The independent components analysis identified a series of spatially independent components (ICs), and subsequent analyses identified ICs whose expression was related to some phase of the task in at least one age group while controlling for gender. Some of the ICs were only related to the task in the young age group. This finding could imply that young adults employ different strategies than old adults for performing the task [44]. However, these young-group-specific ICs have strong correlations with ICs that are task-related to both age groups suggesting they do not represent a young age group-specific functional network.

We next examined functional network connectivity between components. Advanced age was associated with decreased functional network connectivity between all components, in most cases reducing connectivity to non-significant levels as compared to young adults. Mediation analyses determined that poorer task performance (slower speed) was partially the result of age-related decreases in functional network connectivity between IC7 (SMA/precentral) and IC23 (mid. cingulate) and between IC13 (precuneus) and IC24 (mid/sup. frontal cortex). In other words, reduced functional network connectivity between these components was in the causal pathway between increased age and decreased task performance. It is important to point out that although advancing age has broad ranging effects of functional network connectivity, not all of these effects translate to performance decrements. Only the network connections between these two sets of ICs were related to the speeded task performance.

The SMA/precentral component was strongly related to the load effects during retention in both age groups and to load independent effects during stimulus and probe for the young. The SMA/precentral region has been previously identified in verbal working memory tasks and is hypothesized to be involved with sub-vocal rehearsal of verbal information [45], [46], [47], [48]. We previously identified that speeded task performance is particularly sensitive to the gray matter volume in this region [24] raising the question of how sensitive functional connections are to underlying changes in structure. The SMAs' connections to cingulate regions, part of IC 23, have demonstrated an aging effect in a study by Wu and colleagues (2007) [20]. This same study demonstrated a significant negative correlation between the connectivity between these regions and reaction time on a motor task, supporting the alternate idea that these pre-motor regions are simply related to motor slowing [49].

Components 1 and 13 appear to be anterior and posterior parts of the default mode network, as further discussed below. Supporting this idea is the fact that in both age groups these ICs demonstrated task-induced deactivation (TID). And the strength of the TID is greater in the young adults, as previously demonstrated [21]. This change in TID in the older adults combined with the negative correlation between these two ICs may hint at different strategies employed by the two age groups.

The two components 10 and 11 appear to be homologues of each other encompassing pre-frontal cortical regions in the left and right hemispheres, respectively. Interestingly, even though both ICs were task-related, only IC 11 showed strong functional network connections with other ICs which also differed between the age groups. Previous work demonstrated a change in the strength of lateralization of working memory task related activity with advancing age [50]. In that study both young and old adults used left prefrontal brain regions; however, older adults had greater activity in right prefrontal brain regions. In our study, there were age-group differences in functional network connectivity with the right sided pre-frontal cortex, IC 11, and not with the left, IC 10, supporting the idea of advancing age affecting the lateralization of pre-frontal resources.

Interestingly, IC 13 was one of the most interconnected nodes for both age groups, supporting the idea that precuneus and superior parietal regions play important roles in successful performance of the delayed item recognition task [51], [52]. Component 13 includes brain regions often associated with the default mode network [53], [54]. It is visually similar to independent components 50, 53 and 25 derived from 603 participants in the study by Allen et al. [55], which they describe as comprising the default mode network. Our IC 13 has a spatial correlation of 0.64 with the DMN template supplied with the ICA software used for the current study and developed by the same authors. In addition to spatial similarity, the relationship between this component and the task demands was negative, supporting the idea that when attention to the task is required, expression of this component decreases [56], [57], [58]. However, our IC13 has positive functional connectivity values for both age groups with other ICs that have positive relationships and increasing expression with increased task demands. This differs from reports of negative relationships between expression of the DMN and task-positive networks [59]. This suggests that while the areas associated with the DMN do show overall reduced expression with increased task demand, the story is a bit more complicated in that the DMN may be more intricately associated with task performance. The positive functional connection between the DMN and other areas that increase expression with task demand suggests that the brain areas in the DMN may contribute to cognitive processes needed for task performance. Support for this idea is the fact that the strength of functional network connectivity between IC 13 and the prefrontal cortex IC 24 significantly mediates the effect of advancing age on task performance. Therefore, future work into how the DMN and other areas interact during cognitive tasks may eventually yield greater insight into the role this network plays in task performance.

One possible underlying cause for age-related decline in functional connectivity was investigated using a global volumetric measure, normalized whole brain volume (nWBV). Normalized whole brain volume significantly differed between age groups and was related to the functional connectivity strength for all components predictive of task performance. Mediation analyses demonstrated that the age effect on the functional connections related to task performance was not significantly explained by differences in global brain volume. Therefore, advancing age led to decreased brain volume and decreased functional connectivity between brain regions that ultimately affected task performance. However, there was no evidence to support the hypothesis that age-related alterations in functional connectivity are the result of global brain volume changes. Future work will investigate whether regional brain volume measures are better predictors of functional connectivity, as opposed to the gross brain wide measure used here. It is also likely that interruption in the functional connection between brain regions is the result of regional tissue changes or white matter tracts [60]. Diffusion tensor imaging can test whether the density of the white matter tracks physically connecting these brain regions predicts functional connectivity strength.

Advanced age decreased the strength of the relationship between task demands and expression of the ICs. This finding could result from many of the same physiological issues that impact voxel-wise general linear modeling analyses in older adults [19]. Namely, neuro-vascular changes and increased noise in the BOLD signal decrease the detectability of task-related signal changes in older adults. Measures of functional connectivity test the strength of correlation between expressions of different spatial components within participant. Therefore, any global age-related or idiosyncratic effects on an individuals' data will likely affect all of their components. This makes tests of functional connectivity robust to individual physiological differences but still sensitive to the effects of age related increases of noise in the BOLD signal.

The current experiment was conducted within the context of the overall research model of our laboratory [22], [23]. This model posits that age-related changes in task performance might be explained by age-related changes in neural activity, which themselves could result in part from age or AD-related pathological changes in brain structure or blood flow. The current approach, using a global measure of brain volume, did not explain the age related changes in functional connectivity; however, regional volume measure may be more informative in explaining the age effect. Furthermore, the model includes cognitive reserve (CR) which is the partial protection from the negative effects of age due to individual differences. Cognitive reserve might act by moderating the impact of age-related neural changes on task performance and the effect of CR on task performance might be mediated by alterations in neural activity [11]. Although not tested in the current study, the strength of functional connectivity between brain regions may be related to cognitive reserve. Greater cognitive reserve may result in increased functional connectivity, or at least maintained connectivity with advancing age. There is also the possibility that tests for more complex relationships between nodes, or network wide complexity measures [61], [62], [63], may further elucidate the relationships between functional connectivity, cognitive reserve, aging and cognitive performance. These possible associations will be the focus of future research.

The current work uses measures of within-participant functional network connectivity derived from ICA [16], which differs from previous multivariate analyses coming from our laboratory. The adoption of this new technique requires further elaboration. Barring the similarities and differences between the ICA and principal components analysis (PCA) algorithms, the largest difference between previous work and the current approach is the level of the data that the multivariate analyses are applied to and how the design matrices are used. The standard statistical parametric mapping (SPM) approach performs univariate general linear model analyses to the data for all individuals [64]. This results in contrast images for each condition for each individual; in the current situation, these are the load dependent and independent effects during the stimulus, retention and probe task phases. Univariate group analyses typically test within and between group differences for each voxel in the contrast images for each condition. The multivariate linear modeling (MLM) [65] approaches we have previously used rely on identifying the principal components across group means of contrast images within a condition to identify the directions of greatest variance between groups [3]. This usually involves only a few brain images, i.e. typically fewer than 10 and sometimes as few as 2. The ordinal trend analysis canonical variance analysis (OrT CVA) again uses the contrast images from each individual for each condition [66]; however, rather than relying on group mean contrast images, it uses all image for each subject in each condition. For *N* subjects and *T* task conditions there will be (T-1)*N contrast images, thus, subject-level variance plays a much bigger role in ORT CVA than in MLM. In contrast, the ICA analysis used here is applied before any univariate analyses are performed [38], [67]. Therefore, derivation of the spatial ICs does not involve any first-level design matrix, but is entirely data driven and preserves much of the participant level signal variation. The three multivariate techniques lie along a continuum of increased supervised-learning constraints: the least constrained is ICA, which embodies no knowledge about experimental design, whereas OrT-CVA involves some constraints, while still leaving substantial inter-subject variance to drive the analysis. MLM is the most constrained analysis with substantial reduction of the data rank *prior* to the PCA, leaving only very few data points that are submitted to the PCA.

Another point to consider is that we applied the group ICA across young and old age groups, rather than conducting two separate within group analyses. ICA across the young and old groups limits the potential inflation of between-group differences by eliminating the need to match spatial components [68]. A recent paper demonstrated that the ICA methods used here are robust to individual differences in amplitude and translations of spatial components; therefore, individuals do not drive group level derived independent components [69]. Other work has demonstrated that group level ICA is sensitive enough to identify a spatial component present in only 10–15% of the participants [70] and that components specific to a subset of participants do not induce erroneous activation in the rest of the participants [67].

The current work only considers the relationship between the task demands and the components for determining which are task-related and which are not. No further assessment of how task-demands affect the strength of functional connectivity is made. It is plausible that the relationships between task demands and brain activity fluctuate across time or that the strength of the functional connectivity between brain regions fluctuates over time. General linear modeling and the correlation analyses used here average over such fluctuations; however, alternate approaches exist which can investigate the effect of task-demands on connectivity strength. The most straightforward approach is to break up levels of task difficulty into different experimental runs. Then the functional connectivity between brain networks or regions can be assessed for the effects of task demand. A more sophisticated approach uses Kalman filtering to capture load dependent fluctuations in connectivity strength. Work by Kang et al. 2011 and Chang and Glover 2010 demonstrated with resting state data that the strength of the relationships between brain regions varied significantly over time [71], [72]. Another alternative performs principal components analysis on trial averaged data from a working memory task [73]. This approach identified covariance patterns and their averaged temporal progression over the different stages of the memory trials. An interesting extension to their work is to investigate the correlations, as estimate of connectivity, between the networks of brain regions they identified and see how these connections are affected by task demands. Future avenues of research could investigate time varying behavior in the current data for age related differences in brain activity.

To recapitulate, although both age groups used a series of spatial components in a similar manner across task phases, the strength and distribution of functional connectivity between these components differed across the age groups. Differences in the functional connectivity between multiple brain regions significantly explained a portion of the age-related differences in task performance. The differences in functional connectivity between these regions were not explained by age-related changes in global brain volume. These results suggest that age-related differences in the coordination of neural activity between brain regions may partially underlie differences in cognitive performance.

## Supporting Information

Supporting Information S1 (DOCX)
