# Supplementary material for: Age-Related Changes in Task Related Functional Network Connectivity
Source: PLoS One. 2012 Sep 18;7(9):e44421. doi: 10.1371/journal.pone.0044421 (PMC3445529; doi:10.1371/journal.pone.0044421)
Supplement: Supporting Information S1 — (DOCX) [file pone.0044421.s001.docx]

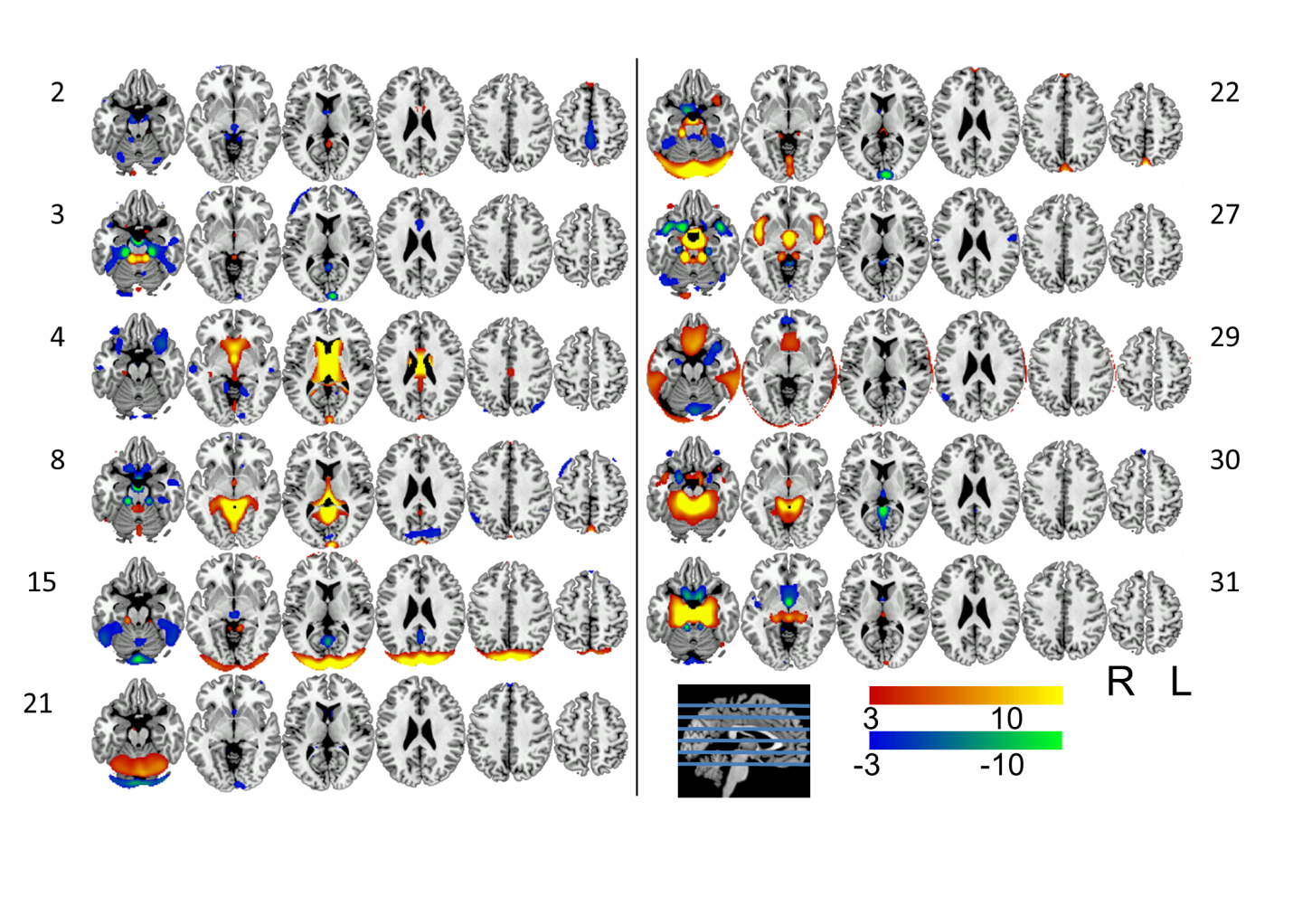


Figure S1. The 11 spatially independent components identified as artifacts. The components are numbered using the arbitrary ordering resulting from the ICA which extracted 36 total components.


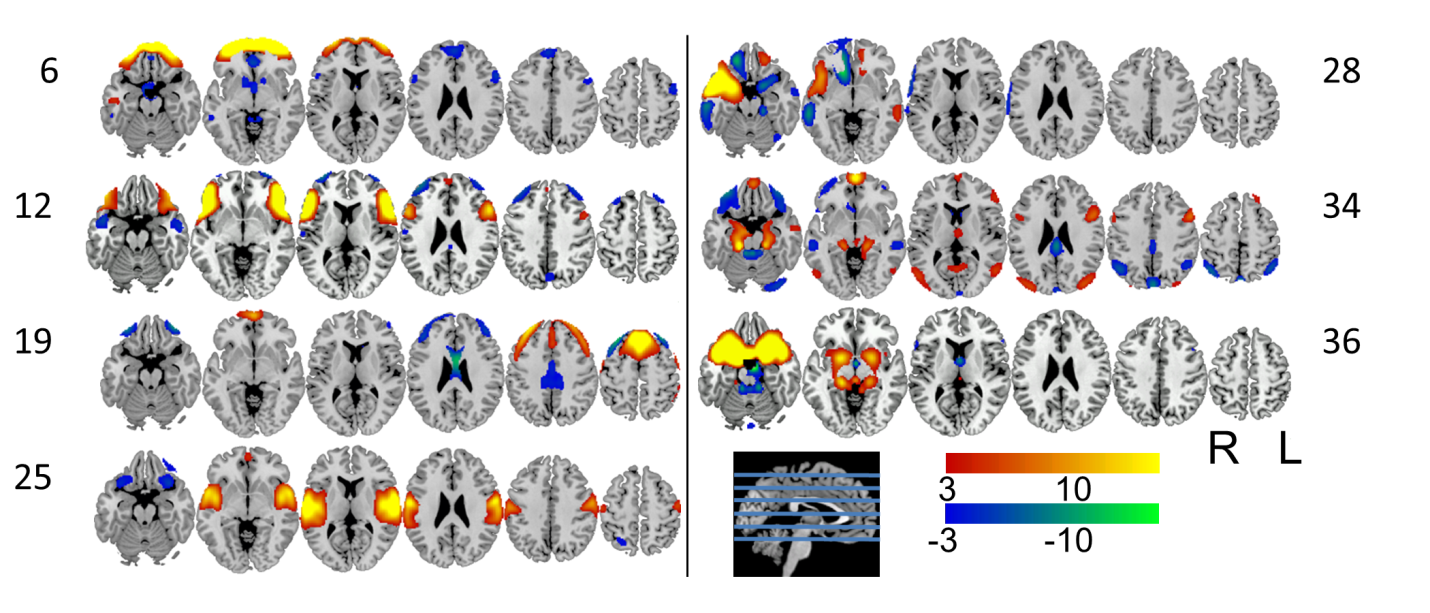


Figure S2. The 7 spatially independent components not related to any aspect of the task. The components are numbered using the arbitrary ordering resulting from the ICA which extracted 36 total components.

| Table S1 Component 1 |  |  |  |  |  |  |  |
| --- | --- | --- | --- | --- | --- | --- | --- |
| Region | Hemi. | B.A. | x | y | z | Z | k |
| *Positive Direction* |  |  |  |  |  |  |  |
| Mid. Frontal Orb. | R | 11 | 0 | 52 | -12 | 60.49 | 9657 |
| -- | R | 11 | -12 | 26 | -4 | 13.05 | -- |
| -- | L | 11 | 12 | 26 | -4 | 10.95 | -- |
| Sup. Temp. Pole | L | 38 | 36 | 14 | -26 | 5.00 | 304 |
| Mid. Temporal Pole | R | 20 | -36 | 16 | -36 | 4.06 | 134 |
| *Negative Direction* |  |  |  |  |  |  |  |
| Mid. Frontal Orb. | L | 10 | 2 | 70 | -6 | 7.67 | 2660 |
| Sup. Frontal Orb. | R | 11 | -28 | 64 | 0 | 7.22 | -- |
| Sup. Frontal Orb. | L | 11 | 24 | 68 | -2 | 6.83 | -- |
| -- | R | -- | -10 | 6 | -24 | 4.54 | 226 |
| Mid. Temporal | L | 21 | 60 | -4 | -20 | 4.26 | 242 |
| -- | - | -- | 0 | -26 | 12 | 4.18 | 126 |
| Mid. Temporal | R | 21 | -56 | -2 | -22 | 3.91 | 129 |
| Inf. Frontal Orb. | L | 47 | 46 | 20 | -4 | 3.89 | 202 |

*Notes:* Height threshold of Z > 3.0 and cluster size > 100. B.A.: Brodmann Areas, Z: Z-scored values of the component maps using the standard deviation across voxels within the map, k: cluster size, -- refers to local maxima or locations without atlas labels.

| Table S2 Component 5 |  |  |  |  |  |  |  |
| --- | --- | --- | --- | --- | --- | --- | --- |
| Region | Hemi. | B.A. | x | y | z | Z | k |
| *Positive Direction* |  |  |  |  |  |  |  |
| Calcarine | L | 17 | 6 | -94 | 0 | 56.43 | 8071 |
| Precuneus | R | -- | -2 | -68 | 64 | 7.9 | 236 |
| Precuneus | R | 5 | -2 | -48 | 74 | 4.56 | -- |
| Mid. Frontal Orb. | R | 10 | -4 | 64 | -6 | 4.45 | 153 |
| *Negative Direction* |  |  |  |  |  |  |  |
| Cerebellar Crus1 | L | -- | 42 | -76 | -28 | 7.47 | 1444 |
| Cerebellar Crus1 | L | -- | 36 | -82 | -26 | 7.33 | -- |
| Cerebellum 3 | R | 30 | -18 | -30 | -22 | 6.41 | 122 |
| Med. Sup. Frontal | L | -- | 0 | 42 | 52 | 6.28 | 371 |
| SMA | L | -- | 0 | 24 | 62 | 3.88 | -- |
| Sup. Temp. Pole | L | -- | 38 | 16 | -18 | 6.14 | 223 |
| Mid. Occipital | L | 19 | 24 | -80 | 40 | 5.74 | 2351 |
| Cuneus | L | -- | 2 | -74 | 30 | 4.97 | -- |
| Cuneus | L | 19 | 12 | -80 | 36 | 4.91 | -- |
| -- | L | -- | 18 | -30 | -50 | 5.48 | 292 |
| -- | L | -- | 12 | -24 | -50 | 5.02 | -- |
| -- | R | -- | -4 | -14 | -30 | 4.81 | -- |
| Mid. Frontal Orb. | L | 47 | 38 | 58 | -12 | 5.18 | 628 |
| Mid. Frontal Orb. | L | -- | 38 | 62 | -2 | 4.79 | -- |
| Cerebellar Crus1 | R | -- | -46 | -64 | -28 | 4.79 | 349 |
| -- | R | -- | -4 | 8 | 8 | 4.60 | 220 |
| Vermis | 3 | -- | 0 | -38 | -8 | 4.51 | 233 |
| Cerebelum 4,5 | R | 30 | -10 | -42 | -10 | 4.35 | -- |
| Cerebelum 4,5 | L | 30 | 10 | -42 | -8 | 4.30 | -- |
| Mid. Frontal Orb. | R | -- | -40 | 52 | -14 | 4.03 | 209 |
| Mid. Frontal Orb. | R | 46 | -44 | 54 | -2 | 3.27 | -- |
| Mid. Frontal Orb. | R | 10 | -38 | 62 | 0 | 3.04 | -- |
| Mid. Temporal | L | 37 | 50 | -68 | 8 | 3.65 | 188 |

*Notes:* Height threshold of Z > 3.0 and cluster size > 100. B.A.: Brodmann Areas, Z: Z-scored values of the component maps using the standard deviation across voxels within the map, k: cluster size, -- refers to local maxima or locations without atlas labels.

| Table S3 Component 7 |  |  |  |  |  |  |  |
| --- | --- | --- | --- | --- | --- | --- | --- |
| Region | Hemi. | B.A. | x | y | z | Z | k |
| *Positive Direction* |  |  |  |  |  |  |  |
| SMA | L | 6 | 0 | -2 | 74 | 47.52 | 6097 |
| Sup. Frontal | R | 6 | -16 | 4 | 72 | 28.94 | -- |
| Precentral | L | 6 | 48 | 2 | 56 | 11.51 | 2295 |
| Mid. Frontal | R | 6 | -48 | 2 | 56 | 10.59 | -- |
| Mid. Frontal | R | -- | -32 | 34 | 50 | 10.54 | -- |
| Mid. Frontal | R | 46 | -36 | 54 | 24 | 5.52 | 201 |
| Mid. Frontal | L | -- | 38 | 56 | 24 | 4.78 | 140 |
| *Negative Direction* |  |  |  |  |  |  |  |
| Mid. Frontal | R | -- | -34 | 10 | 64 | 14.40 | 2956 |
| -- | - | -- | 0 | 38 | 58 | 14.15 | -- |
| Mid. Frontal | L | 8 | 30 | 20 | 62 | 13.81 | -- |
| Mid. Frontal | R | -- | -52 | 16 | 42 | 6.58 | 706 |
| SupraMarginal | R | 40 | -58 | -32 | 50 | 5.87 | -- |
| Precentral | R | 6 | -56 | 2 | 46 | 5.10 | -- |
| -- | L | -- | 14 | -8 | 26 | 6.46 | 139 |
| -- | L | -- | 12 | 2 | 24 | 5.97 | -- |
| -- | R | -- | -12 | -2 | 24 | 6.12 | 126 |
| -- | R | -- | -14 | -10 | 26 | 5.79 | -- |
| Precentral | L | -- | 54 | 14 | 44 | 6.03 | 1072 |
| Mid. Frontal | L | 44 | 52 | 22 | 42 | 5.59 | -- |
| Mid. Frontal | L | 45 | 46 | 34 | 40 | 5.41 | -- |
| Mid. Cingulum | L | -- | 10 | -12 | 34 | 5.82 | 728 |
| -- | L | -- | 8 | -4 | 30 | 5.73 | -- |
| -- | R | -- | -6 | 0 | 28 | 5.55 | -- |
| -- | R | 25 | -8 | 8 | -12 | 4.26 | 171 |

*Notes:* Height threshold of Z > 3.0 and cluster size > 100. B.A.: Brodmann Areas, Z: Z-scored values of the component maps using the standard deviation across voxels within the map, k: cluster size, -- refers to local maxima or locations without atlas labels.

| Table S4 Component 9 |  |  |  |  |  |  |  |
| --- | --- | --- | --- | --- | --- | --- | --- |
| Region | Hemi. | B.A. | x | y | z | Z | k |
| *Positive Direction* |  |  |  |  |  |  |  |
| Calcarine | L | 17 | 0 | -76 | 10 | 21.09 | 13962 |
| Cuneus | L | 18 | 2 | -80 | 24 | 20.70 | -- |
| Lingual | R | 18 | -8 | -66 | 2 | 18.96 | -- |
| *Negative Direction* |  |  |  |  |  |  |  |
| Calcarine | L | 17 | 8 | -98 | 2 | 9.03 | 1607 |
| Mid. Occipital | L | 18 | 30 | -94 | -2 | 5.44 | -- |
| Inf. Occipital | L | 18 | 22 | -98 | -6 | 5.25 | -- |
| Vermis_4 | 5 | -- | 0 | -44 | 4 | 7.23 | 290 |
| -- | L | -- | 6 | -32 | 10 | 5.77 | -- |
| -- | R | -- | -4 | -34 | 6 | 4.89 | -- |
| Inf. Occipital | R | 18 | -26 | -96 | -10 | 5.49 | 907 |
| -- | R | 18 | -32 | -92 | -16 | 5.27 | -- |
| -- | - | -- | 0 | -44 | -28 | 4.70 | 203 |
| Sup. Parietal | L | 7 | 32 | -72 | 56 | 3.67 | 366 |
| Angular | L | 39 | 44 | -70 | 46 | 3.51 | -- |
| -- | L | -- | 52 | -62 | 44 | 3.39 | -- |

*Notes:* Height threshold of Z > 3.0 and cluster size > 100. B.A.: Brodmann Areas, Z: Z-scored values of the component maps using the standard deviation across voxels within the map, k: cluster size, -- refers to local maxima or locations without atlas labels.

| Table S5 Component 10 |  |  |  |  |  |  |  |
| --- | --- | --- | --- | --- | --- | --- | --- |
| Region | Hemi. | B.A. | x | y | z | Z | k |
| *Positive Direction* |  |  |  |  |  |  |  |
| Inf. Parietal | L | -- | 40 | -62 | 54 | 16.22 | 4628 |
| Precuneus | L | 7 | 8 | -78 | 50 | 7.76 | -- |
| Mid. Frontal | L | -- | 36 | 62 | 4 | 12.63 | 9394 |
| Mid. Frontal | L | 44 | 50 | 16 | 40 | 12.53 | -- |
| Mid. Frontal | L | 9 | 42 | 14 | 52 | 11.56 | -- |
| Post. Cingulate | L | 23 | 2 | -36 | 32 | 5.25 | 326 |
| Mid. Frontal | R | 46 | -42 | 56 | 4 | 4.74 | 260 |
| Angular | R | 7 | -36 | -68 | 52 | 4.57 | 446 |
| Angular | R | 39 | -44 | -58 | 54 | 4.28 | -- |
| Cerebellum Crus2 | R | -- | -36 | -70 | -40 | 3.52 | 102 |
| Mid. Temporal | L | 21 | 64 | -40 | -6 | 3.51 | 157 |
| Mid. Temporal | L | 21 | 64 | -28 | -8 | 3.47 | -- |
| *Negative Direction* |  |  |  |  |  |  |  |
| Mid. Frontal Orb. | R | 11 | -4 | 64 | -10 | 6.34 | 1111 |
| Med. Sup. Frontal | L | -- | -2 | 64 | 22 | 5.19 | -- |
| Sup. Frontal | R | 9 | -14 | 58 | 34 | 3.57 | -- |
| Inf. Frontal Orb. | R | 47 | -48 | 26 | -4 | 4.91 | 1058 |
| Mid. Temporal | R | 39 | -50 | -68 | 20 | 4.52 | 608 |
| SMA | R | 8 | -10 | 22 | 66 | 4.43 | 409 |
| Precuneus | R | 23 | -4 | -58 | 26 | 4.40 | 822 |
| Precuneus | R | 5 | -6 | -58 | 60 | 3.88 | -- |
| Cerebellar Crus1 | L | -- | 38 | -84 | -28 | 3.79 | 391 |
| Cerebellum Crus2 | L | -- | 30 | -84 | -32 | 3.61 | -- |

*Notes:* Height threshold of Z > 3.0 and cluster size > 100. B.A.: Brodmann Areas, Z: Z-scored values of the component maps using the standard deviation across voxels within the map, k: cluster size, -- refers to local maxima or locations without atlas labels.

| Table S6 Component 11 | |  |  |  |  |  |  |
| --- | --- | --- | --- | --- | --- | --- | --- |
| Region | Hemi. | B.A. | x | y | z | Z | k |
| *Positive Direction* |  |  |  |  |  |  |  |
| Precentral | R | -- | -52 | 12 | 34 | 17.49 | 8738 |
| Mid. Frontal | R | 45 | -44 | 48 | 4 | 8.72 | -- |
| Mid. Frontal | R | 6 | -36 | 0 | 62 | 7.96 | -- |
| Sup. Occipital | R | 7 | -30 | -74 | 48 | 13.92 | 4122 |
| Inf. Parietal | L | 7 | 32 | -72 | 48 | 6.69 | 813 |
| SMA | R | -- | -2 | 14 | 52 | 6.36 | 664 |
| Inf. Temporal | R | 37 | -52 | -60 | -16 | 5.31 | 849 |
| Inf. Frontal Tri. | L | 45 | 50 | 34 | 26 | 4.50 | 412 |
| *Negative Direction* |  |  |  |  |  |  |  |
| SMA | L | 8 | 10 | 24 | 66 | 6.54 | 979 |
| SupraMarginal | L | 2 | 60 | -32 | 32 | 5.75 | 1946 |
| Mid. Temporal | L | 37 | 54 | -62 | 10 | 4.08 | -- |
| Sup. Frontal | L | 46 | 24 | 58 | 30 | 5.53 | 1102 |
| Med. Sup. Frontal | L | 9 | 4 | 60 | 36 | 3.83 | -- |
| Inf. Frontal Tri. | L | 38 | 54 | 22 | -2 | 4.59 | 869 |
| Inf. Frontal Orb. | L | 47 | 50 | 36 | -4 | 4.13 | -- |
| -- | L | -- | 40 | 14 | -14 | 3.10 | -- |
| Precuneus | L | 5 | 2 | -52 | 56 | 4.49 | 956 |
| Mid. Cingulum | L | 23 | 2 | -22 | 42 | 3.86 | -- |
| Precuneus | L | 7 | 8 | -60 | 68 | 3.62 | -- |

*Notes:* Height threshold of Z > 3.0 and cluster size > 100. B.A.: Brodmann Areas, Z: Z-scored values of the component maps using the standard deviation across voxels within the map, k: cluster size, -- refers to local maxima or locations without atlas labels.

| Table S7 Component 13 | |  |  |  |  |  |  |
| --- | --- | --- | --- | --- | --- | --- | --- |
| Region | Hemi. | B.A. | x | y | z | Z | k |
| *Positive Direction* |  |  |  |  |  |  |  |
| Precuneus | L | -- | 0 | -56 | 22 | 21.41 | 9160 |
| Precuneus | L | -- | 0 | -68 | 36 | 21.06 | -- |
| Mid. Frontal Orb. | L | 10 | 0 | 64 | -8 | 11.26 | 942 |
| Angular | R | 19 | -42 | -74 | 38 | 9.83 | 2248 |
| Angular | L | 39 | 46 | -70 | 36 | 9.22 | 2046 |
| *Negative Direction* |  |  |  |  |  |  |  |
| Cuneus | L | 18 | 4 | -94 | 22 | 8.27 | 342 |
| Mid. Frontal | R | 46 | -38 | 48 | 26 | 5.80 | 1054 |
| Mid. Frontal | L | 46 | 38 | 48 | 30 | 5.60 | 1059 |
| Insula | R | -- | -46 | 10 | -10 | 5.20 | 365 |
| -- | L | -- | 48 | 14 | -8 | 5.02 | 449 |
| -- | L | -- | 30 | -50 | 6 | 4.13 | 158 |
| Calcarine | R | -- | -28 | -54 | 4 | 4.06 | 104 |
| -- | L | -- | 30 | -52 | 72 | 3.50 | 131 |
| Sup. Parietal | L | 7 | 18 | -64 | 70 | 3.19 | -- |
| Inf. Parietal | L | -- | 42 | -52 | 62 | 3.16 | -- |

*Notes:* Height threshold of Z > 3.0 and cluster size > 100. B.A.: Brodmann Areas, Z: Z-scored values of the component maps using the standard deviation across voxels within the map, k: cluster size, -- refers to local maxima or locations without atlas labels.

| Table S8 Component 14 | |  |  |  |  |  |  |
| --- | --- | --- | --- | --- | --- | --- | --- |
| Region | Hemi. | B.A. | x | y | z | Z | k |
| *Positive Direction* |  |  |  |  |  |  |  |
| -- | - | -- | 0 | -26 | 74 | 25.69 | 9285 |
| Med. Sup. Frontal | L | -- | 0 | 42 | 54 | 8.25 | 1085 |
| Sup. Frontal | L | 8 | 22 | 32 | 56 | 4.97 | -- |
| Sup. Frontal | L | 9 | 16 | 40 | 54 | 4.78 | -- |
| Mid. Frontal Orb. | R | 46 | -44 | 50 | -6 | 5.24 | 426 |
| Mid. Frontal Orb. | L | 10 | 38 | 60 | -4 | 4.93 | 298 |
| -- | L | 25 | 2 | 6 | -14 | 3.65 | 206 |
| *Negative Direction* |  |  |  |  |  |  |  |
| -- | - | -- | 0 | -6 | -32 | 8.56 | 751 |
| -- | - | -- | 0 | -14 | -30 | 7.30 | -- |
| Sup. Temp. Pole | L | -- | 20 | 10 | -28 | 3.57 | -- |
| SupraMarginal | R | 40 | -62 | -32 | 42 | 6.27 | 1439 |
| Mid. Frontal | R | 9 | -32 | 42 | 44 | 5.19 | 540 |
| Mid. Frontal | R | 45 | -42 | 48 | 24 | 3.84 | -- |
| Mid. Frontal | R | -- | -32 | 60 | 20 | 3.84 | -- |
| -- | L | -- | 62 | -40 | 44 | 5.13 | 1372 |
| -- | L | -- | 62 | -30 | 48 | 4.88 | -- |
| Sup. Frontal | L | -- | 18 | 14 | 70 | 5.13 | 231 |
| Sup. Frontal | L | 9 | 30 | 46 | 44 | 5.05 | 530 |
| Mid. Frontal | L | -- | 36 | 38 | 46 | 4.96 | -- |
| Precentral | L | 6 | 50 | 8 | 52 | 4.46 | -- |
| -- | L | -- | 16 | -32 | -48 | 4.90 | 140 |
| -- | R | -- | -2 | -24 | -50 | 4.58 | -- |
| -- | R | -- | -10 | -30 | -48 | 4.04 | -- |
| Precentral | R | 6 | -50 | 4 | 52 | 4.45 | 446 |
| Inf. Frontal Oper. | R | 44 | -58 | 12 | 30 | 4.09 | -- |
| Precentral | R | 6 | -60 | 4 | 34 | 3.88 | -- |
| Inf. Frontal Oper. | L | 44 | 60 | 16 | 28 | 4.19 | 190 |
| Sup. Temp. Pole | R | -- | -42 | 14 | -18 | 3.92 | 112 |
| Sup. Temp. Pole | L | 38 | 44 | 16 | -18 | 3.90 | 188 |
| Mid. Frontal | L | -- | 38 | 56 | 24 | 3.77 | 115 |
| Mid. Frontal | L | -- | 32 | 62 | 22 | 3.61 | -- |

*Notes:* Height threshold of Z > 3.0 and cluster size > 100. B.A.: Brodmann Areas, Z: Z-scored values of the component maps using the standard deviation across voxels within the map, k: cluster size, -- refers to local maxima or locations without atlas labels.

| Table S9 Component 16 | |  |  |  |  |  |  |
| --- | --- | --- | --- | --- | --- | --- | --- |
| Region | Hemi. | B.A. | x | y | z | Z | k |
| *Positive Direction* |  |  |  |  |  |  |  |
| Cerebellar Crus1 | R | 19 | -32 | -80 | -20 | 18.50 | 7407 |
| Lingual | L | 18 | 30 | -86 | -16 | 16.62 | 8225 |
| Sup. Parietal | L | 7 | 26 | -70 | 54 | 4.33 | -- |
| Med. Sup. Frontal | L | -- | 0 | 44 | 54 | 5.56 | 176 |
| Precuneus | L | -- | 0 | -56 | 18 | 5.05 | 170 |
| *Negative Direction* |  |  |  |  |  |  |  |
| Calcarine | L | 17 | 4 | -96 | 8 | 9.78 | 1102 |
| -- | L | -- | 2 | -90 | -24 | 5.12 | -- |
| Cerebellum 3 | R | -- | -18 | -28 | -20 | 6.15 | 333 |
| Angular | R | 40 | -46 | -58 | 56 | 3.62 | 158 |
| Inf. Parietal | R | 39 | -56 | -56 | 42 | 3.14 | -- |

*Notes:* Height threshold of Z > 3.0 and cluster size > 100. B.A.: Brodmann Areas, Z: Z-scored values of the component maps using the standard deviation across voxels within the map, k: cluster size, -- refers to local maxima or locations without atlas labels.

| Table S10 Component 17 | |  |  |  |  |  |  |
| --- | --- | --- | --- | --- | --- | --- | --- |
| Region | Hemi. | B.A. | x | y | z | Z | k |
| *Positive Direction* |  |  |  |  |  |  |  |
| Postcentral | L | 2 | 38 | -40 | 66 | 15.76 | 6584 |
| Postcentral | L | -- | 36 | -32 | 68 | 14.81 | -- |
| Postcentral | L | 3 | 48 | -32 | 58 | 14.29 | -- |
| Sup. Parietal | R | -- | -44 | -40 | 60 | 13.85 | 6108 |
| Postcentral | R | -- | -40 | -28 | 64 | 12.77 | -- |
| -- | - | -- | 0 | -14 | -28 | 10.74 | 390 |
| -- | L | -- | 2 | -6 | -32 | 7.12 | -- |
| -- | - | -- | 0 | -10 | -18 | 5.93 | -- |
| SMA | L | 6 | 0 | -16 | 54 | 7.74 | 1170 |
| Sup. Temp. Pole | L | 38 | 38 | 22 | -24 | 4.08 | 108 |
| *Negative Direction* |  |  |  |  |  |  |  |
| SMA | L | 6 | 0 | 0 | 74 | 7.65 | 689 |
| -- | - | -- | 0 | -34 | 78 | 5.90 | -- |
| -- | - | -- | 0 | -20 | 78 | 5.75 | -- |
| Insula | R | -- | -42 | 16 | -2 | 4.59 | 735 |
| Insula | R | -- | -46 | 0 | -2 | 3.52 | -- |
| Precuneus | L | -- | 0 | -74 | 54 | 4.48 | 157 |
| -- | L | -- | 46 | 14 | -2 | 4.31 | 537 |
| Insula | L | -- | 46 | 4 | -2 | 4.19 | -- |

*Notes:* Height threshold of Z > 3.0 and cluster size > 100. B.A.: Brodmann Areas, Z: Z-scored values of the component maps using the standard deviation across voxels within the map, k: cluster size, -- refers to local maxima or locations without atlas labels.

| Table S11 Component 18 | |  |  |  |  |  |  |
| --- | --- | --- | --- | --- | --- | --- | --- |
| Region | Hemi. | B.A. | x | y | z | Z | k |
| *Positive Direction* |  |  |  |  |  |  |  |
| Mid. Temporal | R | 21 | -62 | -32 | -4 | 12.32 | 8602 |
| Mid. Temporal | R | 21 | -60 | -24 | -8 | 12.09 | -- |
| Mid. Temporal | R | 37 | -58 | -60 | 12 | 10.58 | -- |
| Mid. Temporal | L | 21 | 60 | -24 | -8 | 11.09 | 6650 |
| Mid. Temporal | L | 21 | 58 | -54 | 16 | 7.94 | -- |
| Cerebellum 4,5 | L | 30 | 6 | -44 | -8 | 4.81 | 160 |
| Sup. Temp. Pole | L | 38 | 50 | 22 | -10 | 4.66 | 118 |
| Calcarine | L | 18 | 4 | -94 | 10 | 4.59 | 118 |
| SMA | L | 6 | 0 | 18 | 64 | 3.76 | 124 |
| SMA | R | 6 | -2 | 12 | 70 | 3.51 | -- |
| *Negative Direction* |  |  |  |  |  |  |  |
| -- | - | -- | 0 | -56 | 12 | 5.36 | 277 |
| Postcentral | R | 43 | -60 | -4 | 28 | 5.27 | 876 |
| Postcentral | L | 43 | 60 | -2 | 26 | 5.16 | 1420 |
| RolandicOper. | L | -- | 46 | -6 | 16 | 3.57 | -- |
| Insula | L | -- | 44 | 2 | 8 | 3.42 | -- |
| Cerebellar Crus1 | R | -- | -40 | -48 | -38 | 4.66 | 857 |
| Mid. Frontal | R | 9 | -32 | 42 | 38 | 3.96 | 448 |
| Sup. Frontal | R | 8 | -24 | 32 | 50 | 3.48 | -- |
| Sup. Frontal | R | 8 | -24 | 14 | 60 | 3.29 | -- |
| Mid. Frontal | L | 8 | 28 | 30 | 52 | 3.82 | 459 |
| Mid. Frontal | L | 9 | 30 | 36 | 44 | 3.75 | -- |

*Notes:* Height threshold of Z > 3.0 and cluster size > 100. B.A.: Brodmann Areas, Z: Z-scored values of the component maps using the standard deviation across voxels within the map, k: cluster size, -- refers to local maxima or locations without atlas labels.

| Table S12 Component 20 | |  |  |  |  |  |  |
| --- | --- | --- | --- | --- | --- | --- | --- |
| Region | Hemi. | B.A. | x | y | z | Z | k |
| *Positive Direction* |  |  |  |  |  |  |  |
| Precuneus | R | -- | 0 | -66 | 62 | 28.84 | 10084 |
| Sup. Parietal | R | 40 | -38 | -54 | 62 | 5.63 | -- |
| Mid. Occipital | L | 19 | 36 | -82 | 30 | 5.11 | -- |
| -- | L | -- | 2 | -88 | -18 | 7.62 | 472 |
| Cerebellar Crus1 | L | 19 | 50 | -66 | -24 | 6.42 | 1016 |
| Sup. Temp. Pole | L | 38 | 56 | 14 | -6 | 5.47 | 364 |
| Mid. Frontal | R | 9 | -36 | 40 | 36 | 5.29 | 645 |
| Sup. Frontal | R | 6 | -30 | 0 | 66 | 5.19 | 300 |
| SupraMarginal | L | 40 | 62 | -32 | 34 | 4.94 | 439 |
| Sup. Temp. Pole | R | 38 | -52 | 14 | -10 | 4.55 | 254 |
| Sup. Frontal | L | 6 | 28 | 2 | 66 | 4.08 | 154 |
| Mid. Frontal | L | 46 | 34 | 48 | 34 | 3.66 | 122 |
| *Negative Direction* |  |  |  |  |  |  |  |
| Postcentral | R | 43 | -58 | -8 | 28 | 4.56 | 1468 |
| Postcentral | R | -- | -50 | -18 | 54 | 4.38 | -- |
| Inf. Occipital | R | 18 | -26 | -96 | -6 | 4.47 | 805 |
| Mid. Occipital | L | 18 | 30 | -94 | -4 | 4.29 | 520 |
| Postcentral | L | 43 | 58 | -6 | 30 | 4.22 | 1244 |
| Postcentral | L | 3 | 46 | -20 | 58 | 4.06 | -- |

*Notes:* Height threshold of Z > 3.0 and cluster size > 100. B.A.: Brodmann Areas, Z: Z-scored values of the component maps using the standard deviation across voxels within the map, k: cluster size, -- refers to local maxima or locations without atlas labels.

| Table S13 Component 23 | |  |  |  |  |  |  |
| --- | --- | --- | --- | --- | --- | --- | --- |
| Region | Hemi. | B.A. | x | y | z | Z | k |
| *Positive Direction* |  |  |  |  |  |  |  |
| Mid. Cingulum | L | 32 | 0 | 16 | 42 | 16.46 | 8383 |
| -- | - | 23 | 0 | -30 | 30 | 6.25 | -- |
| Precuneus | R | -- | 0 | -46 | 52 | 4.59 | -- |
| -- | R | -- | -50 | 16 | -6 | 10.42 | 907 |
| Inf. Frontal Orb. | L | 38 | 50 | 18 | -6 | 9.22 | 718 |
| Mid. Frontal | R | 46 | -30 | 54 | 26 | 6.26 | 828 |
| Mid. Frontal | L | 46 | 32 | 54 | 28 | 5.86 | 655 |
| Precuneus | L | 7 | 2 | -76 | 42 | 5.13 | 433 |
| Precentral | R | 6 | -52 | 6 | 38 | 4.32 | 169 |
| SupraMarginal | R | -- | -60 | -42 | 28 | 3.81 | 138 |
| *Negative Direction* |  |  |  |  |  |  |  |
| -- | - | 25 | 0 | 18 | 2 | 8.09 | 795 |
| -- | - | -- | 0 | -2 | -8 | 5.08 | -- |
| Caudate | R | -- | -14 | 26 | 4 | 4.77 | -- |
| -- | - | -- | 0 | -88 | -22 | 6.75 | 343 |
| -- | L | -- | 28 | -46 | 6 | 6.58 | 1347 |
| Hippocampus | L | -- | 20 | -38 | 10 | 6.45 | -- |
| -- | L | 17 | 24 | -54 | 22 | 3.57 | -- |
| -- | R | 37 | -28 | -50 | 2 | 6.28 | 1178 |
| -- | R | -- | -20 | -38 | 10 | 6.15 | -- |
| -- | R | -- | -14 | -34 | 14 | 5.75 | -- |
| SMA | R | 6 | -12 | 22 | 68 | 4.85 | 207 |
| Sup. Frontal | R | -- | -24 | 4 | 72 | 4.16 | -- |
| Sup. Frontal | R | 6 | -26 | -10 | 74 | 3.60 | -- |
| Calcarine | L | 17 | 4 | -96 | 10 | 4.75 | 130 |
| Cuneus | L | 18 | 4 | -92 | 24 | 4.39 | -- |
| Sup. Frontal | L | -- | 14 | 26 | 66 | 4.19 | 139 |
| Precentral | L | 6 | 24 | -12 | 76 | 4.01 | -- |
| Sup. Frontal | L | -- | 16 | 16 | 70 | 3.86 | -- |
| Mid. Occipital | R | 39 | -44 | -72 | 30 | 4.04 | 567 |
| Inf. Frontal Orb. | R | -- | -46 | 50 | -4 | 3.98 | 138 |
| -- | L | 7 | 20 | -68 | 66 | 3.96 | 1326 |
| Angular | L | 39 | 48 | -66 | 26 | 3.80 | -- |
| Precuneus | R | -- | -2 | -68 | 64 | 3.74 | -- |
| Mid. Frontal | R | 9 | -42 | 20 | 50 | 3.89 | 119 |
| Postcentral | L | 43 | 60 | -6 | 28 | 3.40 | 167 |
| RolandicOper. | L | -- | 42 | -6 | 16 | 3.17 | -- |

*Notes:* Height threshold of Z > 3.0 and cluster size > 100. B.A.: Brodmann Areas, Z: Z-scored values of the component maps using the standard deviation across voxels within the map, k: cluster size, -- refers to local maxima or locations without atlas labels.

| Table S14 Component 24 | |  |  |  |  |  |  |
| --- | --- | --- | --- | --- | --- | --- | --- |
| Region | Hemi. | B.A. | x | y | z | Z | k |
| *Positive Direction* |  |  |  |  |  |  |  |
| Med. Sup. Frontal | L | -- | 0 | 44 | 52 | 22.88 | 11087 |
| Sup. Frontal | R | 9 | -14 | 44 | 52 | 18.50 | -- |
| Sup. Frontal | L | 9 | 16 | 44 | 52 | 16.41 | -- |
| Angular | R | 39 | -48 | -66 | 46 | 7.98 | 1728 |
| Angular | R | 39 | -50 | -68 | 36 | 7.76 | -- |
| Rectus | L | 11 | 0 | 54 | -16 | 5.36 | 196 |
| Inf. Parietal | L | -- | 52 | -58 | 50 | 4.84 | 462 |
| Angular | L | 39 | 54 | -62 | 38 | 4.22 | -- |
| -- | L | -- | 62 | -36 | 48 | 3.22 | -- |
| Precuneus | R | 5 | -2 | -56 | 70 | 4.68 | 104 |
| *Negative Direction* |  |  |  |  |  |  |  |
| Precuneus | L | 7 | 10 | -74 | 48 | 6.30 | 2603 |
| Inf. Parietal | L | 7 | 30 | -68 | 42 | 5.81 | -- |
| Inf. Parietal | L | 40 | 42 | -46 | 48 | 3.48 | -- |
| Sup. Frontal Orb. | L | 11 | 22 | 66 | -4 | 6.00 | 671 |
| Mid. Frontal Orb. | R | 11 | -10 | 68 | -4 | 4.99 | 340 |
| SMA | L | -- | 0 | 4 | 68 | 4.82 | 158 |
| Sup. Occipital | R | 7 | -26 | -68 | 40 | 4.76 | 1128 |
| -- | L | 25 | 2 | 22 | 0 | 4.52 | 313 |
| -- | L | -- | 14 | 28 | -2 | 3.93 | -- |
| Inf. Frontal Tri. | L | 47 | 36 | 26 | 0 | 4.45 | 954 |
| Inf. Frontal Oper. | L | -- | 46 | 14 | 24 | 4.29 | -- |
| Inf. Frontal Oper. | R | 44 | -44 | 6 | 26 | 3.55 | 101 |

*Notes:* Height threshold of Z > 3.0 and cluster size > 100. B.A.: Brodmann Areas, Z: Z-scored values of the component maps using the standard deviation across voxels within the map, k: cluster size, -- refers to local maxima or locations without atlas labels.

| Table S15 Component 26 | |  |  |  |  |  |  |
| --- | --- | --- | --- | --- | --- | --- | --- |
| Region | Hemi. | B.A. | x | y | z | Z | k |
| *Positive Direction* |  |  |  |  |  |  |  |
| Med. Sup. Frontal | L | -- | 0 | 64 | 2 | 29.12 | 12543 |
| Sup. Frontal | R | 10 | -18 | 68 | 4 | 17.19 | -- |
| Sup. Frontal | L | 11 | 18 | 68 | 2 | 16.26 | -- |
| Caudate | R | -- | -12 | 24 | 8 | 4.88 | 476 |
| Caudate | L | -- | 6 | 18 | 8 | 4.70 | -- |
| -- | L | -- | 14 | 26 | 6 | 4.58 | -- |
| *Negative Direction* |  |  |  |  |  |  |  |
| -- | L | -- | 40 | 56 | -16 | 10.71 | 2554 |
| Mid. Frontal Orb. | R | -- | -42 | 52 | -16 | 9.22 | -- |
| -- | R | -- | -30 | 56 | -18 | 8.62 | -- |
| -- | - | -- | 0 | 42 | 56 | 7.48 | 701 |
| Mid. Frontal | R | 9 | -38 | 12 | 62 | 4.96 | -- |
| Mid. Frontal | R | 6 | -42 | 2 | 62 | 4.74 | -- |
| -- | L | -- | 38 | 14 | 62 | 5.01 | 380 |
| Mid. Frontal | L | 8 | 34 | 22 | 60 | 4.90 | -- |
| Precentral | L | -- | 40 | 4 | 64 | 4.40 | -- |
| Sup. Occipital | R | 7 | -30 | -66 | 42 | 3.27 | 112 |
| Angular | R | 40 | -36 | -56 | 42 | 3.18 | -- |

*Notes:* Height threshold of Z > 3.0 and cluster size > 100. B.A.: Brodmann Areas, Z: Z-scored values of the component maps using the standard deviation across voxels within the map, k: cluster size, -- refers to local maxima or locations without atlas labels.

| Table S16 Component 32 | | | | | | | |
| --- | --- | --- | --- | --- | --- | --- | --- |
| Region | Hemi. | B.A. | x | y | z | Z | k |
| *Positive Direction* |  |  |  |  |  |  |  |
| -- | R | -- | -10 | 4 | -22 | 51.83 | 9432 |
| ParaHippocampal | L | 28 | 12 | 0 | -22 | 47.95 | -- |
| Sup. Temp. Pole | L | 38 | 34 | 14 | -26 | 26.27 | -- |
| *Negative Direction* |  |  |  |  |  |  |  |
| -- | R | -- | -2 | -14 | -26 | 18.22 | 1602 |
| Cerebellum 3 | R | 30 | -18 | -32 | -26 | 16.91 | -- |
| -- | - | -- | 0 | -10 | -18 | 14.33 | -- |
| Sup. Temp. Pole | L | -- | 44 | 8 | -12 | 13.75 | 2960 |
| -- | R | -- | -4 | 20 | 2 | 6.74 | -- |
| -- | L | -- | 36 | 36 | -20 | 5.89 | -- |
| Sup. Temp. Pole | R | -- | -44 | 12 | -14 | 13.47 | 2071 |
| Inf. Frontal Orb. | R | 47 | -32 | 36 | -20 | 5.75 | -- |
| -- | R | -- | -26 | 2 | -8 | 3.86 | -- |
| -- | - | -- | 0 | -26 | -2 | 7.25 | 276 |
| Vermis | 3 | -- | 2 | -36 | -4 | 4.44 | -- |
| Fusiform | L | 36 | 36 | -4 | -38 | 4.75 | 243 |
| Med. Sup. Frontal | L | -- | 0 | 44 | 50 | 4.64 | 173 |
| Cerebellum Crus2 | L | -- | 2 | -86 | -30 | 4.30 | 108 |

*Notes:* Height threshold of Z > 3.0 and cluster size > 100. B.A.: Brodmann Areas, Z: Z-scored values of the component maps using the standard deviation across voxels within the map, k: cluster size, -- refers to local maxima or locations without atlas labels.

| Table S17 Component 33 | | | | | | | |
| --- | --- | --- | --- | --- | --- | --- | --- |
| Region | Hemi. | B.A. | x | y | z | Z | k |
| *Positive Direction* |  |  |  |  |  |  |  |
| Mid. Temporal | L | 21 | 60 | -4 | -16 | 18.71 | 5389 |
| Sup. Temp. Pole | L | 38 | 48 | 14 | -16 | 15.83 | -- |
| Mid. Temporal | R | 21 | -60 | -6 | -14 | 13.52 | 3455 |
| Rectus | L | -- | 0 | 62 | -16 | 8.79 | 596 |
| -- | - | -- | 0 | -20 | -48 | 6.25 | 127 |
| Precuneus | L | -- | 2 | -74 | 56 | 5.05 | 220 |
| Precentral | L | 6 | 40 | 2 | 60 | 3.87 | 251 |
| Precentral | L | -- | 46 | -10 | 58 | 3.34 | -- |
| Precentral | L | -- | 42 | -18 | 64 | 3.05 | -- |
| Precentral | R | 6 | -44 | -6 | 58 | 3.69 | 220 |
| Precentral | R | -- | -52 | -10 | 44 | 3.36 | -- |
| *Negative Direction* |  |  |  |  |  |  |  |
| -- | L | -- | 40 | 62 | -2 | 15.79 | 2497 |
| Mid. Frontal Orb. | R | 46 | -44 | 54 | 0 | 12.26 | 2323 |
| -- | L | 19 | 32 | -88 | -24 | 5.26 | 326 |
| Fusiform | R | 20 | -30 | -6 | -40 | 5.06 | 490 |
| SupraMarginal | L | 2 | 64 | -28 | 34 | 4.93 | 669 |
| SupraMarginal | R | 40 | -60 | -38 | 34 | 4.78 | 1364 |
| SupraMarginal | R | 40 | -58 | -40 | 46 | 4.12 | -- |
| Inf. Parietal | R | 40 | -50 | -54 | 52 | 4.08 | -- |
| -- | R | -- | -16 | 6 | -18 | 3.89 | 138 |

*Notes:* Height threshold of Z > 3.0 and cluster size > 100. B.A.: Brodmann Areas, Z: Z-scored values of the component maps using the standard deviation across voxels within the map, k: cluster size, -- refers to local maxima or locations without atlas labels.

| Table S18 Component 35 | |  |  |  |  |  |  |
| --- | --- | --- | --- | --- | --- | --- | --- |
| Region | Hemi. | B.A. | x | y | z | Z | k |
| *Positive Direction* |  |  |  |  |  |  |  |
| Mid. Temporal Pole | L | 20 | 38 | 14 | -30 | 24.14 | 7370 |
| Mid. Temporal | L | 21 | 54 | 2 | -26 | 19.78 | -- |
| Inf. Temporal | R | 36 | -36 | 4 | -40 | 13.72 | 3772 |
| Mid. Frontal Orb. | R | 10 | -6 | 50 | -8 | 4.37 | 232 |
| -- | L | -- | 36 | -50 | -38 | 3.96 | 243 |
| Cerebellum 4,5 | L | 37 | 28 | -40 | -30 | 3.12 | -- |
| *Negative Direction* |  |  |  |  |  |  |  |
| Cerebellum 3 | R | 30 | -16 | -30 | -22 | 13.50 | 4633 |
| -- | - | -- | 0 | -14 | -30 | 11.32 | -- |
| -- | R | -- | -2 | -22 | -48 | 10.26 | -- |
| Mid. Temporal | L | 20 | 60 | -24 | -12 | 6.42 | 1345 |
| Mid. Temporal | L | 20 | 62 | -32 | -10 | 6.26 | -- |
| Inf. Temporal | L | 20 | 54 | -48 | -26 | 3.27 | -- |
| Cerebellum 4,5 | L | 30 | 16 | -32 | -18 | 6.06 | 215 |
| Cerebellum 4,5 | L | 30 | 10 | -40 | -8 | 3.44 | -- |
| Cerebellar Crus1 | R | -- | -44 | -62 | -32 | 5.19 | 740 |
| Cuneus | L | 18 | 2 | -96 | 16 | 5.11 | 100 |
| Cuneus | L | 18 | 2 | -92 | 28 | 3.35 | -- |
| -- | L | 45 | 62 | 22 | 10 | 4.47 | 446 |
| -- | L | 43 | 66 | 2 | 16 | 3.68 | -- |
| -- | L | 43 | 68 | -12 | 20 | 3.23 | -- |

*Notes:* Height threshold of Z > 3.0 and cluster size > 100. B.A.: Brodmann Areas, Z: Z-scored values of the component maps using the standard deviation across voxels within the map, k: cluster size, -- refers to local maxima or locations without atlas labels.
